# Proportion of the CD19-Positive and CD19-Negative Lymphocytes and Monocytes within the Peripheral Blood Mononuclear Cell Set Is Characteristic for Rheumatoid Arthritis

**DOI:** 10.3390/medicina55100630

**Published:** 2019-09-24

**Authors:** Irina Kholodnyuk, Anda Kadisa, Simons Svirskis, Sabine Gravelsina, Peteris Studers, Irina Spaka, Alina Sultanova, Sandra Lejniece, Aivars Lejnieks, Modra Murovska

**Affiliations:** 1 Institute of Microbiology and Virology, Riga Stradins University, LV-1067 Riga, Latvia; anda.kadisa@rsu.lv (A.K.); simons.svirskis@rsu.lv (S.S.); sabine.gravelsina@rsu.lv (S.G.); irina.spaka@inbox.lv (I.S.); alina.sultanova@rsu.lv (A.S.);; 2 Department of Internal Diseases, Riga Stradins University, LV-1038 Riga, Latvia; sandra.lejniece@rsu.lv (S.L.); aivars.lejnieks@rsu.lv (A.L.); 3 Inter-Department Laboratory of Traumatology and Orthopedics, Riga Stradins University, LV-1013 Riga, Latvia; peteris.studers@rsu.lv; 4 Riga East University Hospital, LV-1038 Riga, Latvia

**Keywords:** rheumatoid arthritis, osteoarthritis, B lymphocytes, T lymphocytes, monocytes, CCR1, CCR2

## Abstract

*Background and objectives:* Composition of the peripheral blood (PB) cell populations and their activation state reflect the immune status of a patient. Rheumatoid arthritis (RA) is characterized by abnormal B- and T-cell functions. The objective of this study was to assess the profiles of the PB mononuclear cell (PBMC) populations in patients with rheumatoid and osteoarthritis (OA) in comparison with healthy control (HC) subjects in order to evaluate the PBMC profiles as a potential diagnostic characteristic in RA. The second aim was to assess the CCR1 and CCR2 expression on PB lymphocytes and correlate it with the plasma levels of matrix metallopeptidase 9 (MMP-9), IL-17F, TNF-α, IL-6, and IL-10. *Materials and Methods:* The frequency and phenotype, including CCR1 and CCR2, of the PBMC populations (monocytes, CD19^+^B cells, and T/NK lymphocytes) in RA (*n* = 15) and OA (*n* = 10) patients and HC (*n* = 12) were analyzed by five-color flow cytometry. DNA of the viruses, HHV-6, HHV-7, and B19, in the whole blood and cell-free plasma, were assessed by nested-polymerase chain reaction (PCR). *Results:* Active persistent or acute infections, caused by HHV-6, HHV-7, or B19, were not detected in patients of this study. Both CCR1 and CCR2 were determined on the PB B and T/NK lymphocytes in several RA and OA patients and HCs. However, in patients, the frequency of the CCR1-positive T/NK lymphocytes showed a weak negative correlation with the IL-10 level, while the frequency of the CCR2-positive B cells correlated positively with the level of IL-6. Statistically significant differences in the proportions of the CD19-positive and CD19-negative lymphocyte and monocyte subsets within the PBMC set were determined between RA and OA patients and HC adults. *Conclusions:* We have shown in our pilot study with rather small cohorts of patients that the PBMC-population profiles were very consistent, and statistically significantly differed between RA and OA patients and HC subjects.

## 1. Introduction

Rheumatoid arthritis (RA) is an autoimmune chronic inflammatory disease characterized by massive infiltration of synovial tissue with immune cells, mainly macrophages and lymphocytes. These cells are implicated in the degradation of the cartilage and erosion of juxta-articular bone [1]. Numerous proinflammatory cytokines, chemokines, and growth factors have been detected in the RA synovium [2,3]. RA is featured by impaired T- and B-cell functions and an abnormal immune response [4]. Clinical and animal studies disclosed the multiple roles of B cells in the development and severity of RA: aberrant antigen presentation and production of autoantibodies and inflammatory cytokines, such as tumor necrosis factor-α (TNF-α) and interleukin 6 (IL-6) [5]. Aberrant antigen presentation by B cells contributes to chronic T-cell activation [6,7]. Moreover, rheumatoid factor (RF) expressing B cells are accumulated within the RA synovium [8].

One of the mechanisms to recruit leucocytes in synovial tissues relates to the chemotactic gradient, the interaction of chemokines produced in the inflamed synovium, and chemokine receptors expressed on the immune cells [8,9]. Targeting of the chemotactic proteins has been suggested as potential treatment in RA [10]. In preclinical studies, the blockade of numerous chemokines and their receptors, using antibodies, peptide, and small molecule inhibitors, had given promising results. However, most of the human RA trials failed [3]. By contrast, in the proof-of-principle study, the blockade of the chemokine receptor 1 (CCR1) inhibited macrophage infiltration in the synovium of RA patients [11]. Notably, in mouse models, the collagen-induced arthritis was accelerated and worsened when the chemokine receptor 2 (CCR2) was genetically knockout or blocked with specific antibodies [12]. Chemokines and their receptors regulate the lymphopoiesis and hematopoiesis, leukocyte migration, and adhesion. CCR1 share the protein sequence similarity with CCR2. These two receptors belong to the family of the inflammatory chemokine receptors and respond to the same chemokines [13,14]. Both CCR1 and CCR2 are abundantly expressed on PB monocytes, but are inducible in T and B lymphocytes [14,15].

The neutrophil-to-lymphocyte ratio (NLR) is identified as a systemic inflammation marker and has been associated with inflammatory activity and prognosis in many disorders, including a variety of cancers [16] and chronic inflammatory disorders like systemic lupus erythematosus, primary Sjögren’s syndrome, ankylosing spondylitis (AS), OA, and RA [17,18,19]. Interestingly, NLR was not affected by gender, age, or the used drugs (biologics, steroids, sulfasalazine, methotrexate, or leflunomide) in patients with RA (*n* = 136) and AS (*n* = 140) [17]. Two recent studies reported the association between the monocyte–lymphocyte ratio (MLR) and disease activity in RA; the total lymphocyte count was significantly lower in RA patients relative to the OA and HC groups and correlated with the erythrocyte sedimentation rate (ESR), C-reactive protein (CRP) level, and disease activity score of 28 joints (DAS28) [19,20].

The composition of the PB cell populations and their activation state reflects the immune status of a patient and may be characteristic for the RA disease. Nowadays, the clinical flow cytometry (FC) analyses are the routine tests in the evaluation of the immune status of a patient. Proportions between the populations of monocytes and B lymphocytes, the key actors in RA, can be measured without additional manipulations. The aim of this study was to assess, by flow cytometry, the composition of PBMC populations, monocytes, and CD19-positive and CD19-negative lymphocytes, in patients with RA and OA in comparison with HC adults in order to estimate the PBMC population profile as the potential diagnostic characteristic in RA. The second aim of the study was to assess the cell-surface expression of CCR1 and CCR2 in PB lymphocytes of RA and OA patients in comparison with the healthy individuals and correlate the CCR1/CCR2-positivity with the disease.

Several reports demonstrated previously that the ubiquitous blood-cell born human viruses, herpesviruses HHV-6 and HHV-7 and parvovirus B19 (B19V), may become reactivated under immunosuppression conditions, as the treatment with immunosuppressive drugs [21], contributing further to the development of autoimmune diseases, including RA [22,23,24]. To avoid the influence of the HHV-6, HHV-7, or B19V infection re-activation on the immune system, only patients, as well as HC subjects, without persistent infections in the active phase or possible acute infections were included in the study.

## 2. Materials and Methods

### 2.1. Patients and Healthy Control Volunteers

The current pilot flow-cytometry based study was carried out within the Latvian National Research Program in Biomedicine. Patients were randomly selected from the RA and OA patient cohorts of the National Research Program. In this report, only patients with the full set of clinical and laboratory analyses were included. Patients with RA (*n* = 15) and OA (*n* = 10) were recruited from the Riga East University Hospital (REUH, Riga, Latvia) and from the Hospital of Traumatology and Orthopedics (Riga, Latvia) during the period 2012–2015. RA was diagnosed according to the 2010 American College of Rheumatology/European League Against Rheumatism (ACR/EULAR) Classification Criteria [25]. OA patients fulfilled the ACR criteria for the OA affected joints [26]. Clinical parameters such as disease duration, medication, age, sex, DAS28, ESR, CRP, rheumatoid factor (RF), anti-cyclic citrulinated peptide antibodies (anti-CCP) levels, and magnetic resonance imaging (MRI) staging (only for RA patients) were collected. The levels of CRP and ESR in PB were used as indicators of the RA and OA activity; the CRP level >8.0 mg/L and ESR >30.0 mm/h were considered as elevated and corresponded to the active disease [27]. Clinical data of patients are shown in Table 1.

Healthy control (HC) individuals were selected from among local staff volunteers. The EDTA anti-coagulated peripheral blood samples and the plasma samples were frozen at aliquots and stored at −80 °C until the analyses. Ethics approval No 17 (Nr. E-9 (2)) was given on 27 September 2012 by the Riga Stradins University (RSU) Ethics Committee and written informed consent was received from participating patients.

Detection of HHV-6, HHV-7, and B19V genomic sequences in the whole blood and the cell-free plasma DNA was carried out by nested polymerase chain reaction (PCR) with virus-specific primers, as described previously [23,24,28]. Blood plasma samples were tested for the presence of IgM and IgG antibodies to B19V using enzyme immunoassay kits (Mikrogen recomWell, Martinsried, Germany) and according to the manufacturer’s protocols.

### 2.2. Assessment of the MMP-9 and Cytokines Concentrations in the Plasma

The levels of matrix metallopeptidase 9 (MMP-9), interleukin 17F (IL-17F), TNF-α, IL-6, and interleukin 10 (IL-10) in the plasma of patients were measured using enzyme-linked immunosorbent assay (ELISA) kits: MMP-9 (USCNK Life Science Inc., USA), IL-17F (IBL International GmbH, Hamburg, Germany), TNF-α, IL-6, and IL-10 (Biorbyt Ltd., Cambridge, UK).

### 2.3. Cell Frequency and Phenotype Analyses

The EDTA anti-coagulated PB (100 µL) was stained with fluorochrome-conjugated antibodies according to the standard protocol “Direct Immunofluorescence Staining of Whole Blood Using a Lyse/Wash Procedure” (BD Biosciences) in the same day when the samples were collected. Patients with RA (*n* = 15) and OA (*n* = 10), and 12 apparently healthy control individuals, were analyzed by five-color flow cytometry (FC). For the analyses of the frequency and phenotype of each cell population, the following fluorochrome-conjugated mouse anti-human monoclonal antibodies (all from BD Biosciences) were used: CD45-FITC or CD45-Alexa Fluor 700 (clone HI30), CD10-PE (clone HI10a), CD19-PE-Cy5 or CD19-FITC (clone HIB19), CD38-Horizon V450 (clone HB8), CD191-Alexa Fluor 647 (clone 53504), and CD192-Alexa Fluor 647 (clone 48607, specific for both CCR2 variants). CD191-Alexa Fluor 647 and CD192-Alexa Fluor 647 were used for the assessment of the CCR1 and CCR2 cell-surface expression. Cells were acquired on a BD FACSAriaII (BD; Becton, Dickinson and Company, Franklin Lakes, NJ, USA) and analyzed using Diva6.2 software (BD Biosciences). After the selection of the CD45^+^ leukocytes, the granulocyte (CD10^+^), monocyte, and lymphocyte populations were separated by gating on SSC versus FSC and SSC versus CD10-PE plots (Figure S1). CD19^–^CD10^–^ lymphocytes were designed as T/NK lymphocytes.

### 2.4. Statistical Analyses

Statistical analyses and graphs were done using the GraphPad Prism software version 8.0 for Mac (GraphPad Software, San Diego, CA, USA). Normality of numerical data was checked by the Anderson–Darling, D’Agostino and Pearson, and Shapiro–Wilk normality tests. Before the comparison of groups by one-way analysis of variance (ANOVA), the homogeneity of variances was checked using the Brown–Forsythe and Bartlett’s tests. When data were not normally distributed, the non-parametric one-way ANOVA on ranks or Kruskal–Wallis (KW) test followed by the two-stage step-up method of Benjamini, Krieger, and Yekutieli as the post-hoc test was applied. Comparison of two groups was performed by the nonparametric Mann–Whitney (MW) test. The difference between groups was also assessed by the qualitative approach, using the Chi-square (Chi^2^) test, to compare proportions of measurements. Mean levels of the all clinical and laboratory parameters, including the plasma cytokines and MMP-9, were expressed as medians with dispersion, characterized by the interquartile region (IQR). Distributions of the cytokines levels were mostly lognormal and, therefore, were also summarized in natural units as geometric means with geometric standard deviation (SD). Associations between hematological, immunological, and clinical parameters were assessed using the Spearman’s correlation analysis. A *p*-value less than 0.05 (*p* < 0.05) was considered as a statistically significant difference.

## 3. Results

### 3.1. Clinical Characteristics of the Patients

The study included 15 RA patients (12 females and 3 males) with the mean age of 56.5 years (range: 26–80), 10 OA patients (6 females and 4 males) with the mean age of 64.7 years (range: 35–77), and 12 healthy control (HC) volunteers (8 females and 4 males) with the mean age of 56.5 years (range: 25–76). RA patients in the study differed in the disease duration, medication, activity, and aggressiveness (Table 1). However, most of the RA patients (9 out of the 15) were classified as patients with severe disease activity (DAS28 score ≥ 5.1), 5 patients were with moderate disease activity (DAS28 score 3.2–5.1), and only 1 patient had low disease activity (DAS28 score ≤ 3.2), according to the EULAR classification criteria [25].

**Figure 1:**
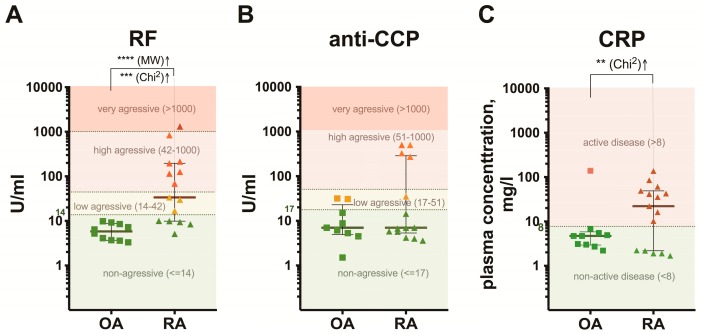
Levels of rheumatoid factor (RF), anti-cyclic citrulinated peptide antibodies (anti-CCP), and C-reactive protein (CRP) in the peripheral blood plasma from rheumatoid arthritis (RA) (*n* = 15) and osteoarthritis (OA) (*n* = 10) patients. Distribution of the RF (**A**), anti-CCP (**B**), and CRP (**C**) values among the individual patients is shown. The reference intervals are as follows: for the RF level, 14.0, 42.0, and 1,000.0 U/mL; for the anti-CCP level, 17.0, 51.0, and 1,000.0 U/mL; for the CRP level, 8.0 mg/L. Data are presented as scatter plots with the medians and interquartile ranges (IQR: 25% percentile and 75% percentile); ** *p* < 0.01, *** *p* < 0.001, **** *p* < 0.0001; MW—Mann–Whitney test, Chi^2^—Chi-square test.

The levels of RF and anti-CCP antibody in the PB plasma were determined as indicators of the disease aggressiveness. The level of RF, with or without anti-CCP data, was used for the RA diagnosis. The RF level >14.0 U/mL and anti-CCP level >17.0 U/mL were considered as elevated [25,27]. The RA patients with RF ≤14.0 U/mL were classified as patients with a non-aggressive course of disease (5 out of the 15 RA patients in our study), with RF between 14.0 and 42.0 U/mL—with low-aggressive RA (3 patients), with RF > 42.0 ≤ 1,000.0 U/mL—with high-aggressive (6 patients), and with RF >1,000.0 U/mL—with very aggressive RA (1 patient). All OA patients in the study had RF ≤14.0 U/mL. By the anti-CCP level, the clinical course of RA was classified into three groups: non-aggressive disease with anti-CCP ≤17.0 U/mL (9 out of the 15 patients), low-aggressive disease with anti-CCP > 17.0 ≤ 51.0 U/mL (2 patients), and high-aggressive RA with anti-CCP > 51.0 ≤ 1,000.0 U/mL (4 patients) [23,25]. By the anti-CCP level, OA patients in the study fall into two groups: non-aggressive disease with anti-CCP ≤17.0 U/mL (8 OA patients) and low-aggressive disease with anti-CCP > 17.0 ≤ 51.0 U/mL (2 OA patients). No significant differences (*p* > 0.05) in the anti-CCP levels were found between the patient groups. Distribution of the RF, anti-CCP, and CRP values among the individual patients is shown on the plots (Figure 1).

We detected statistically significant increases in the concentration values of the cytokines IL-6, IL-10, and IL-17F in the PB plasma of RA patients, when compared with the OA patients (Figure S2). The median TNF-α concentration was also higher in RA (12.5 pg/mL, *n* = 15) than in OA (4.0 pg/mL, *n* = 10) patients, but the difference was not statistically significant between these small cohorts. No significant differences (*p* > 0.05) in the MMP-9 plasma levels were found between the patient groups (Figure S2).

### 3.2. HHV-6, HHV-7, and Parvovirus B19 (B19V) Infection Status

To characterize the type of the HHV-6, HHV-7, and B19V infection, the following criteria were considered [23,24]: persistent infection in active phase (active infection), if the viral DNA sequences are detected in DNA extracted from the cell-free blood plasma and in DNA extracted from the whole blood (plasma DNA-positive and blood DNA-positive); persistent infection in latent phase (latent infection), if the viral DNA sequences are detected only in DNA extracted from the whole blood (plasma DNA-negative and blood DNA-positive); without infection or past infection (plasma DNA-negative and blood DNA-negative).

In the case of B19V, the presence of the virus-specific IgM and IgG antibodies in the blood samples has been also accounted for [24]: persistent infection in active phase, when the sample is the plasma DNA-positive, IgM-negative, and IgG-positive; possible acute infection, when the sample is the plasma DNA-positive, IgM-positive, and IgG-negative; persistent infection in latent phase (latent infection), when the sample is the blood DNA-positive, plasma DNA-negative, IgM-negative, and IgG-positive or the blood DNA-positive, plasma DNA-negative, IgM-negative, and IgG-negative; past infection, when the sample is the blood DNA-negative, plasma DNA-negative, IgM-negative, and IgG-positive; without infection, when the sample is the blood DNA-negative, IgM-negative, and IgG-negative.

In our rather small cohort study, the HHV-6, HHV-7, and B19V persistent infection in active phase or possible acute infection were not found in patients and in HC subjects. Most of the RA and OA patients, and the of all HCs, were without infection or had the past infection status. Persistent infection in the latent phase (latent infection) was determined for HHV-6 in one RA patient only; for B19V, in three RA and four OA patients; for HHV-7, in eight RA and nine OA patients; and did not correlate with the clinical characteristics or levels of cytokines and chemokine receptors (CCR1 and CCR2).

### 3.3. Expression of CCR1 and CCR2 on Peripheral Blood Lymphocytes

**Figure 2:**
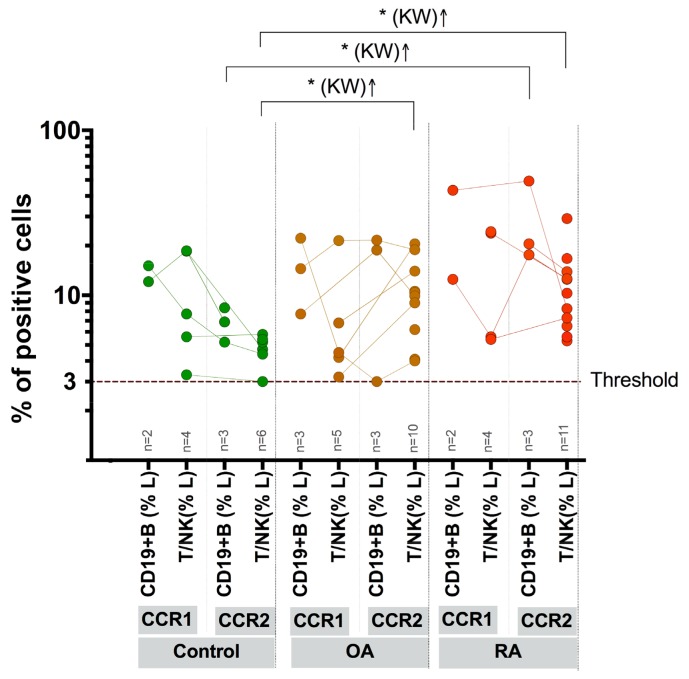
Frequency of the CCR1- and CCR2-positive cells within the CD19^+^B and T/NK lymphocytes in healthy controls and patients with RA and OA. Percentage ≥3.0 of the fluorochrome-stained cells within the gate was considered as percentage of the positive cells; the threshold line was based on staining obtained with the control isotype-matched antibodies (0 < 3.0% of the cells). Data are collected from two independent experiments with two tubes per experiment. The percentage of positive cells is averaged and the mean is shown; SD was within 10% of the mean value in all cases. The collected results are based on at least 100 cells showing the immunophenotype. Data are presented as the comparative paired scatter plot: lines connect the respective individuals; * *p* < 0.05; KW, Kruskal–Wallis test.

The cell-surface expression of CCR1 and CCR2 (≥3.0% of the fluorochrome-positive cells within the gate) on the PB lymphocytes was observed in HC individuals (Figure 2). In two HC subjects, both CCR1 and CCR2 were detected on CD19^+^B cells. Another one HC person was also the B-cell CCR2-positive. Altogether, the mean percentage of the CCR1-positive B cells (in two HC subjects) was 13.6% (range: 12.1%–15.1%), and the mean percentage of CCR2-positive B cells (in three HC subjects) was 6.8% (range: 5.2%–8.4%). On T/NK lymphocytes, CCR1 was expressed in five HCs (the mean of the positive cells was 10.7%; range: 3.3%–18.6%), and CCR2 was expressed in 5 out of the 12 HC persons (the mean was 5.1%; range: 3.3%–18.6%). In OA patients (Figure 2), both CCR1 and CCR2 were detected on B cells in three patients with the mean percentage of the positive cells being 14.8% (range: 7.7%–22.2%) and 14.5% (range: 3.0%–21.6%), respectively. On T/NK lymphocytes, CCR1 was expressed in five OA patients (the mean of the positive cells was 8.0%; range: 3.2%–21.5%), but CCR2 was expressed in all 10 patients (the mean was 10.8%; range: 4.0%–20.5%). In 2 out of the 15 RA patients (Figure 2), both CCR1 and CCR2 were expressed on the PB B cells: 12.5% and 43.3% of B cells were CCR1-positive, and 17.6% and 49.3% were CCR2-positive. Another one RA patient was also B-cell CCR2-positive (20.5%). Notably, these three RA patients had high RF levels: 196.0, 214.4, and 839.2 U/mL, respectively. On T/NK lymphocytes, CCR1 was expressed in four RA patients (the mean of the positive cells was 14.8%; range: 5.4%–24.3%), but CCR2 was expressed in 11 out of the 15 RA patients (the mean of the positive cells was 11.7%; range: 5.3%–29.2%).

Although the numbers of the B-cell CCR1-positive and B-cell CCR2-positive persons did not differ between the cohorts, the frequency (the mean %) of the CCR1- and CCR2-positive B cells was considerably higher in RA patients, 2.1- and 4.3-fold, respectively, relative to the OA and HC groups. The CCR1 expression on T/NK lymphocytes showed a negative correlation with the IL-10 level in plasma (nonparametric Spearman’s *p* = −0.4663, *p* (two-tailed) = 0.0331). In turn, the presence of CCR2 on B lymphocytes positively correlated with the IL-6 level in plasma (Pearson’s *p* = −0.4475, *p* (two-tailed) = 0.0419). Other clinical and laboratory characteristics did not show any correlation with the expression of CCR1 or CCR2 on lymphocytes in PB of RA and OA patients.

### 3.4. Frequency of the Peripheral Blood Mononuclear Cell Populations

**Figure 3:**
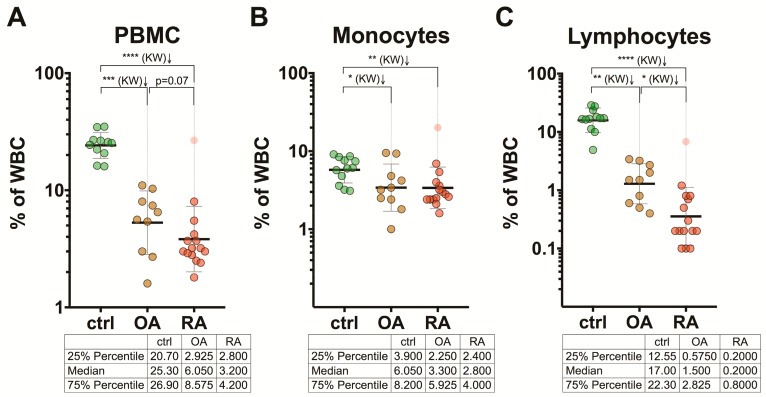
Frequency of the peripheral blood mononuclear cell (PBMC) populations within the white blood cell (WBC) set in patients with RA (*n* = 15) and OA (*n* = 10) and healthy controls (*n* = 12). Distribution of the percentages of PBMC (**A**), monocytes (**B**), and lymphocytes (**C**) among the individual subjects is shown. Data are presented as scatter plots with the geometric mean and geometric standard deviation (SD); * *p* < 0.05, ** *p* < 0.01, *** *p* < 0.001, **** *p* < 0.0001; KW, Kruskal–Wallis test. In the tables, data are presented as the medians and IQR (25% percentile and 75% percentile).

PB mononuclear cells (PBMC) in HCs accounted for 25.3% (the median) of white blood cells (WBCs). However, in patients, the proportion of the PBMC set within the WBCs (leukocytes) was statistically significantly lower: 6.1% in OA patients and 3.2% in RA patients (Figure 3A), also indicating a possible increase of the granulocyte numbers in patients compared with HCs. The difference in the proportion of monocytes within the entire WBC set between the OA and RA groups was not definitive (Figure 3B). However, the lymphocyte numbers in OA and RA patients were significantly lower than in the HC group and accounted for 1.5% (the median) with the range from 0.6% to 2.8% (25% percentile to 75% percentile) for the OA group, 0.2% (the median) with the range from 0.2% to 0.8% for the RA patients, versus 17.0% (the median) with the range from 12.6% to 22.3% for the HC adults (Figure 3C).

**Figure 4:**
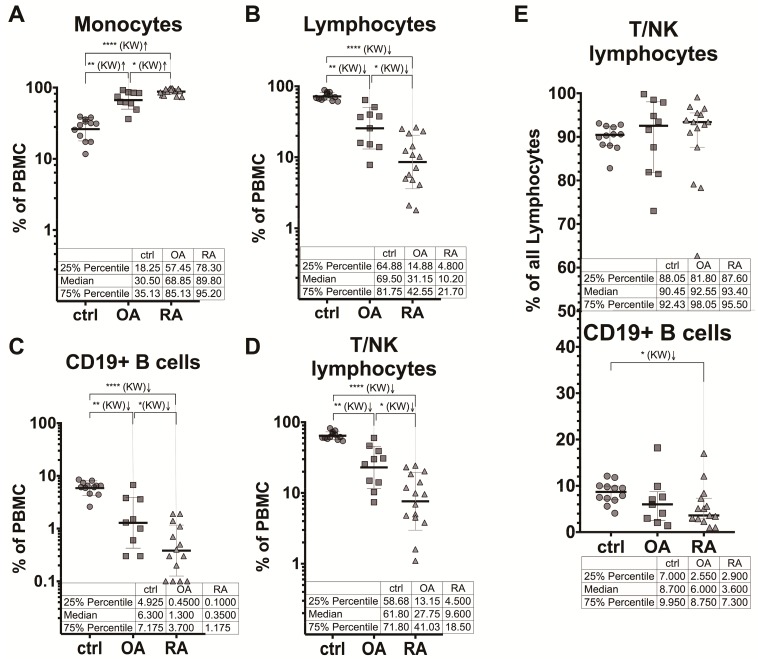
Frequency of the PBMC populations within the PBMC set in patients with RA (*n* = 15) and OA (*n* = 10) and healthy controls (*n* = 12). Distribution of the percentages of monocytes (**A**), lymphocytes (**B**), CD19^+^B cells (**C**), and T/NK lymphocytes (**D**) among the individual subjects is shown; (**E**) presents the proportion of T/NK lymphocytes (**D**) and CD19^+^B cells (**C**) within the lymphocyte set. Data are presented as scatter plots with the geometric means and geometric SD (**A**–**D**) or the medians with IQR (**E**); * *p* < 0.05, ** *p* < 0.01, **** *p* < 0.0001; KW, Kruskal–Wallis test. In the tables, data are presented as the medians and IQR (25% percentile and 75% percentile).

Nevertheless, the proportion of monocytes within the PBMC set was statistically significantly higher both in RA and OA patients (the median was 89.8% and 68.9%, respectively) compared with the HCs with the median of 30.5% (Figure 4A). Consequently, the proportion of lymphocytes, including CD19^+^B cells (CD45^+^CD14^−^CD10^−^CD19^+^) and non-B (CD45^+^CD14^−^CD10^−^CD19^−^), designed as T/NK lymphocytes, was decreased (Figure 4B–D) and accounted (the median % of the cells) for 10.2% and 31.2% in the RA and OA groups, respectively, relative to 69.5% in the HC group. The majority of the lymphocyte population represented the T/NK lymphocytes: 90.5% (the median) in HCs, 92.6% in OA, and 93.4% in RA (Figure 4E). The median frequencies of the T/NK and CD19^+^B cells within the lymphocyte set did not differ significantly between the groups, although the proportions of B cells were decreased in RA and OA patients: 3.6% and 6.0% relative to 8.7% in HCs (Figure 4E). Of note is that the frequency of B cells in the PBMC set of RA patients was very low; 0.35% (the median) compared with 1.30% in OA patients and 6.30% in HCs, and these differences were statistically significant (Figure 4C). Of note is that the RA patients in our study were not receiving the B-cell depletion treatment (as rituximab) and only two patients were treated with methotrexate (MTX) and two with leflunomide (LEF) (Table 1). Both MTX and LEF inhibit the reproduction of lymphocytes, especially B cells [29]. However, in all RA patients, including those treated with nonsteroidal anti-inflammatory drugs (NSAIDs) only, the frequency of B cells as well as T/NK lymphocytes was statistically significantly decreased in comparison with healthy individuals (Figure 4C,D).

**Figure 5:**
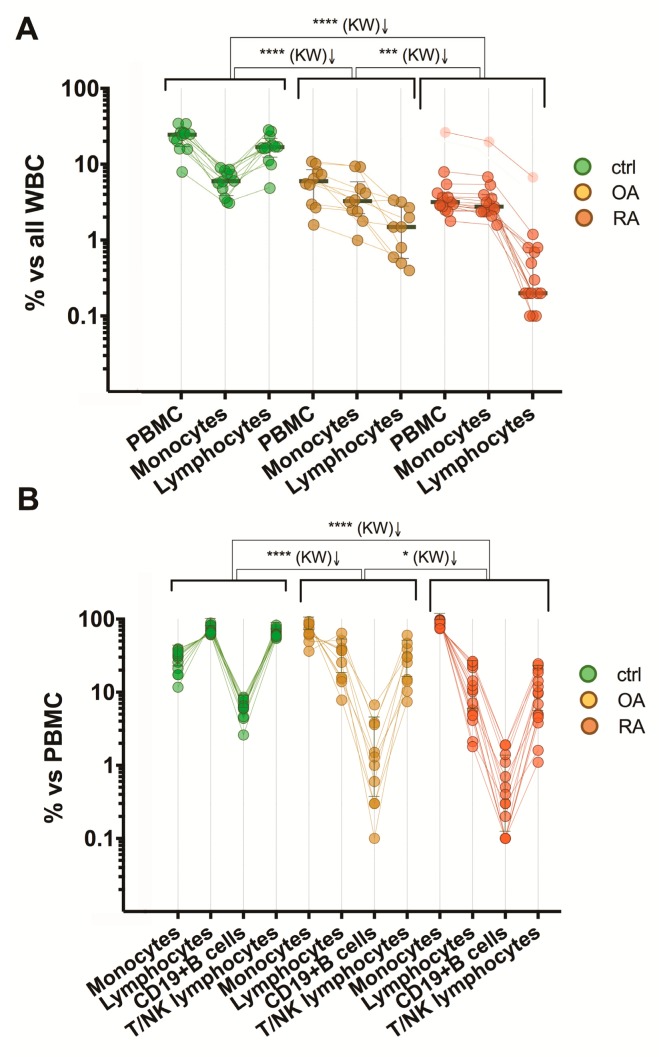
The PBMC-population profiles of patients with RA (*n* = 15) and OA (*n* = 10) in comparison with the healthy control group (*n* = 12). Data show the percentages of the populations within the WBC set (**A**) and PBMC set (**B**) of the individual subjects from three groups. Results are presented as comparative paired scatter plots, where lines connect the respective individuals; * *p* < 0.05, *** *p* < 0.001, **** *p* < 0.0001; KW, Kruskal–Wallis test.

It is noteworthy that 15 RA patients in the study differ significantly in manifestation of the disease; from the low and moderate disease activity with DAS28 score ≤3.2 and between 3.2 and 5.1 (*n* = 1 and *n* = 5, respectively), until the severe disease activity with DAS28 score >5.1 (*n* = 9); from the non-aggressive and low-aggressive course of the disease with RF ≤14.0 U/mL and between 14.0 and 42.0 U/mL (*n* = 5 and *n* = 3, respectively), until the high-aggressive and very aggressive RA with the RF levels between 42.0 and 1,000.0 U/mL and >1,000.0 U/mL (*n* = 6 and *n* = 1, respectively). However, the profile of the PBMC populations in RA patients was maintained to be invariable and statistically significantly differed from that in OA patients and healthy subjects (Figure 5).

## 4. Discussion

One of the first changes observed in RA patients is an increase of the monocyte/macrophage numbers in affected joints. Synovial macrophages are effector cells in the pathogenesis of RA and produce many different inflammatory cytokines, including TNF-α, IL-1, IL-6, and the proteolytic enzymes [30]. TNF-α stimulates the production of inflammatory cytokines and impaired activation of T lymphocytes that promote further development of inflammation [2]. IL-17 mediates the maturation of immune cells, which leads to the exacerbated inflammation of joints in RA patients [31]. In concordance with the published data, we detected statistically significant increases of the cytokines IL-6, IL-10, IL-17F, and TNF-α in the PB plasma of RA patients, if compared with the OA patients (Figure S2).

The percentages of monocytes and lymphocytes within the WBC set, defined for the HC group in our study, were 6.05% (median) with the range from 3.90% to 8.20% (25% percentile to 75% percentile) and 17.00% (median) with the range from 12.55% to 22.30%, respectively (Figure 3B,C), and these values correspond to or fall within the ranges of the reference intervals for the leukocyte subsets in healthy adults reported by others. Of note is that in a healthy population, the gender-, age-, and race-related changes in PB cell counts have been well documented [32,33]. In one large FC analysis of 608 healthy German adults [32], the reference intervals for the PBMC subsets within the WBCs were defined as follows: for monocytes, the median was 0.263 (range: 0.109–0.553) cells in 10^9^/L for males and 0.232 (0.107–0.439) cells in 10^9^/L for females, which accounted for 4.65% (3.2%–6.1%) and 4.24% (3.0%–5.0%), respectively; for lymphocytes, the median was 1.523 (range: 0.692–2.832) cells in 10^9^/L for males and 1.601 (0.737–2.897) cells in 10^9^/L for females, which accounted for 26.96% (20.2%–31.4%) and 29.30% (20.5%–32.6%), respectively. In the same study, the proportion of B cells within the lymphocyte subset was defined as the median of 12.0% (range: 10.0%–16.2%) for males and 13.0% (9.2%–17.5%) for females.

In RA, monocyte/macrophage numbers are increased in the synovium of the affected joints and these numbers correlated with the clinical course of the disease [30]. Our results showed that the proportion of monocytes within the WBC set was decreased about two-fold in both RA and OA groups relative to the age-matched HC group (Figure 3B), which can indicate a dislocation of monocytes towards the inflammation sites. In our study, we demonstrated that within the PBMC set, the proportions of monocytes were significantly higher and the proportions of lymphocytes were significantly lower in both RA and OA patient groups compared with HCs.

In one recent study [19], the markedly increased monocyte–lymphocyte ratio (MLR) was observed in RA patients (*n* = 222) and in OA patients (*n* = 78) compared with the healthy adults (*n* = 170). Because leucopenia and lymphopenia are often detected in many systemic autoimmune rheumatic diseases (SARDs), the authors concluded that the significant increase of MLR in SARDs may be attributed to the lymphocyte number decrease. However, in another recent study [20], the lymphocyte–monocyte ratio (LMR) and the lymphocyte count were significantly lower in RA patients (*n* = 66) relative to the HC/OA groups (*n* = 131 and *n* = 163, respectively). In both of these studies, the total counts of monocytes and lymphocytes were used. Our results showed that in both the RA and OA groups, the proportion of monocytes within the WBCs was decreased about two-fold compared with the age-matched HCs. Although, the frequency of lymphocytes within the WBC set varied between the RA and OA groups, 0.2% and 1.5%, respectively, versus 17% in the HCs (Figure 3B,C).

The more pronounced differences in the proportions of monocytes and lymphocytes between the RA and OA patient groups and relative to the HC group were observed within the PBMC sets. The median percentages of monocytes and lymphocytes in the PBMC set for HCs were 30.5% and 69.5%, respectively (Figure 4A,B). The increase of the monocyte and decrease of the lymphocyte numbers in the PBMC set were higher for RA patients, and accounted (as the median % of the cells in the set) for 89.8% and 10.2%, respectively. For OA patients, the values were 68.9% and 31.2%, which represents the intermediate stage.

Two above-mentioned reports [19,20] demonstrated that the increased MLR or decreased LMR were associated with the RA disease activity. In our study, 15 RA patients differed significantly in the disease duration, medication, activity (from low till severe), and aggressiveness (from non-aggressive till very aggressive). However, we did not find any correlation of the monocyte or lymphocyte frequency within the WBC or PBMC set with other clinical or laboratory characteristics of RA patients, probably owing to the small sample size in our study.

Our results indicated B-cell lymphopenia in both RA and OA patients, with the pronounced decrease of the CD19^+^B lymphocyte numbers in RA and OA patients relative to HCs. The frequency of B cells in the PBMC set of RA patients was very low, 0.35% (the median) compared with 1.30% in OA patients and 6.30% in HCs, and these differences were statistically significant (Figure 4C). It is noteworthy that two groups reported that the IL-10-producing B cells (10^+^Breg) were reduced in RA patients and negatively correlated with the DAS28 activity score [34,35]. In an animal model of RA, chimeric mice lacking the B cells producing IL-10 developed severe collagen-induced arthritis with greatly decreased IL-10-secreting CD4 Treg cells and increased Th1 and Th17 cells [36].

Previously, CCR1 and CCR2 were reported to be expressed on the majority of the PB monocytes in healthy individuals—on approximately 87% and 84% of monocytes, respectively. However, expression of these receptors on PB monocytes in RA patients was significantly lower—on approximately 56% and 76% of monocytes, respectively [9,37]. Notably, only 17% of monocytes from the synovial fluid of RA patients expressed CCR1, and 24% expressed CCR2 [37]. Such an expression pattern of CCR1 and CCR2 suggests that these receptors are involved in monocyte recruitment from the circulation and their retention in the RA joints. CCR1 and CCR2 have been extensively analyzed in T cells and monocytes/macrophages in chronic inflammatory diseases; however, the role of these receptors in B cells is largely unknown [13,14]. In the present study, we determined the cell-surface expression of CCR1 and/or CCR2 on the PB lymphocytes, CD19^+^B, and T/NK, in HC individuals and in patients with RA and OA. However, the frequency (mean %) of the CCR1- and CCR2-positive B cells in RA patients was considerably higher, 2.1- and 4.3-fold, respectively, relative to the OA and HC groups. We also found that the CCR1 expression on T/NK lymphocytes showed negative correlation with the IL-10 level in plasma, whereas the presence of CCR2 on B lymphocytes positively correlated with the IL-6 plasma level. Notably, the correlation between the IL-6 level and the more active and aggressive course of RA has been reported in several studies [38].

To exclude the influence of the HHV-6, HHV-7, or B19V infection re-activation on the immune system, the only subjects without persistent infections in active phase or possible acute infections were included in the study. Therefore, 15 patients with RA, 10 patients with OA, and 12 HC adults remained in this study. Owing to the small sample size, the results of our present study are relatively reliable. Therefore, the larger study is needed to confirm our preliminary, but significant results.

## 5. Conclusions

We have shown that despite the diverged manifestation of the RA disease, the PBMC population profiles were consistent and statistically significantly differed between RA and OA patients and HC subjects. The data obtained in this pilot study are preliminary. To evaluate, if the PBMC population profile could indicate the course of the RA disease and predict the treatment outcome, the prospective comprehensive association study is required.

We also found that both CCR1 and CCR2 were detected on the PB B and T/NK lymphocytes in several RA and OA patients and in some HC subjects. However, in patients, the frequency of the CCR1-positive T/NK lymphocytes showed a weak negative correlation with the IL-10 plasma level, while the frequency of the CCR2-positive B cells correlated positively with the level of IL-6. The association between the individual immune status and the CCR1 and/or CCR2 expression on the PB lymphocytes, including B cells, should be assessed in future studies.

## Figures and Tables

**Table 1:** Clinical characteristics of the patients with rheumatoid arthritis and osteoarthritis.

	Osteoarthritis (*n* = 10) ^1^	Rheumatoid Arthritis (*n* = 15)
DAS28, score		
Mean ± SD	–	5.1 ± 1.5
Range	–	1.1–7.5
ESR, mm/h		
Median (IQR: Q1–Q3)	–	33.5 (17.8–45.0)
Range	–	14.0–60.0
CRP, mg/L		
Median (IQR: Q1–Q3)	4.7 (2.9–5.8)	22.1 (2.2–48.8)
Range	2.2–138.8	1.7–137.1
RF, U/mL		
Median (IQR: Q1–Q3)	5.8 (3.7–8.7)	33.7 (9.9–196.0)
Range	3.3–9.8	5.1–1313.0
anti–CCP, U/mL		
Median (IQR: Q1–Q3)	7.0 (4.9–22.9)	7.0 (5.3–286.7)
Range	1.5–31.6	3.6–500.0
Disease duration, months		
Median (IQR: Q1–Q3)	60.0 (24.0–81.0)	24.0 (9.0–120.0)
Range	6.0–120.0	2.0–480.0
MRI, stage (I–IV)		
Median (IQR: Q1–Q3)	–	2.0 (1.5–3.0)
Range	–	1–4
Treatment ^2^, no. of patients		
NSAIDs	10	10
MTX	–	1
LEF	–	2
Steroids	–	1
SSZ, MTX, Steroids	–	1

DAS28, disease activity score, the number of swollen joints out of the 28; ESR, erythrocyte sedimentation rate; CRP, C-reactive protein; anti-CCP, anti-cyclic citrulinated peptide antibodies; RF, rheumatoid factor; MRI, magnetic resonance imaging stage; NSAIDs, nonsteroidal anti-inflammatory drugs; DMARDs, disease-modifying anti-rheumatic drugs used for the treatment of patients; MTX, methotrexate; LEF, leflunomide; SSZ, sulfasalazine. ^1^ in the analyses, “–”, below the reference interval or not applied; ^2^ for the treatment, “–”, not used; SD, standard deviation; IQR, interquartile range; Q1 and Q3, the first and the third quartiles.

## Supplementary Materials

The following are available online at https://www.mdpi.com/1010-660X/55/10/630/s1. Figure S1: Flow cytometry analysis. Dot-plots of the representative experiments. Figure S2: Levels of MMP-9, TNF-α, IL-6, IL-10, and IL-17F in the peripheral blood plasma of RA and OA patients.
