# Supplementary material for: Proportion of the CD19-Positive and CD19-Negative Lymphocytes and Monocytes within the Peripheral Blood Mononuclear Cell Set Is Characteristic for Rheumatoid Arthritis
Source: Medicina (Kaunas). 2019 Sep 24;55(10):630. doi: 10.3390/medicina55100630 (PMC6843217; doi:10.3390/medicina55100630)
Supplement: Supplementary file 1 [file medicina-55-00630-s001.pdf]

medicina

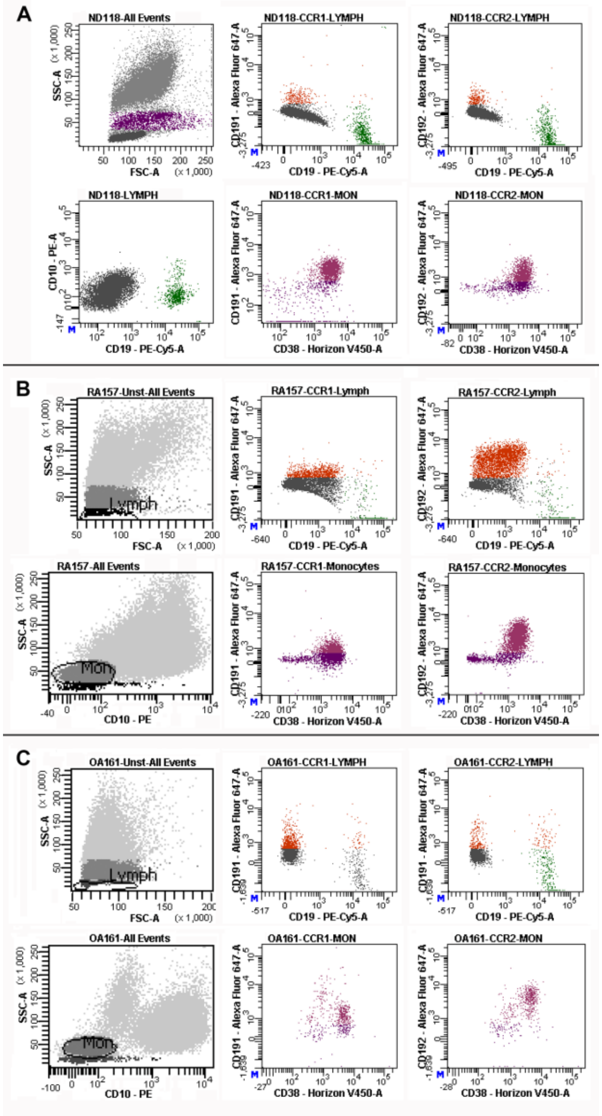

Figure S1. Flow cytometry analysis. Dot-plots of the representative experiments are shown. (**A**) The healthy control individual is the B-cell CCR1- and CCR2-negative and contains 3.3% and 3.0% CCR1- and CCR2-presenting T/NK lymphocytes, respectively; (**B**) the RA patient contains 12.5% and 5.6% of CCR1-expressing B and T/NK lymphocytes and 17.6% and 12.5% of CCR2-expressing B and T/NK lymphocytes, respectively; (**C**) the OA patient is also CCR1- and CCR2-positive of B and T/NK cells, -14.6% and 21.5%, and 21.6% and 18.9%, respectively. Dot plots show the CD10-PE, CD191-Alexa Fluor 647 (CCR1), CD192-Alexa Fluor 647 (CCR2), CD19-PE-Cy5, and CD38-Horizon V450 stained PB leukocytes. A cell population was defined as positive, when ≥3.0% of the cells were stained; the threshold was based on quadrant encompassing the control isotype-matched stained cells (0.0% < 3.0% of the cells). At least 100 events were considered to define the positivity. Dot plots are representative of one experiment with two tubes in parallel.

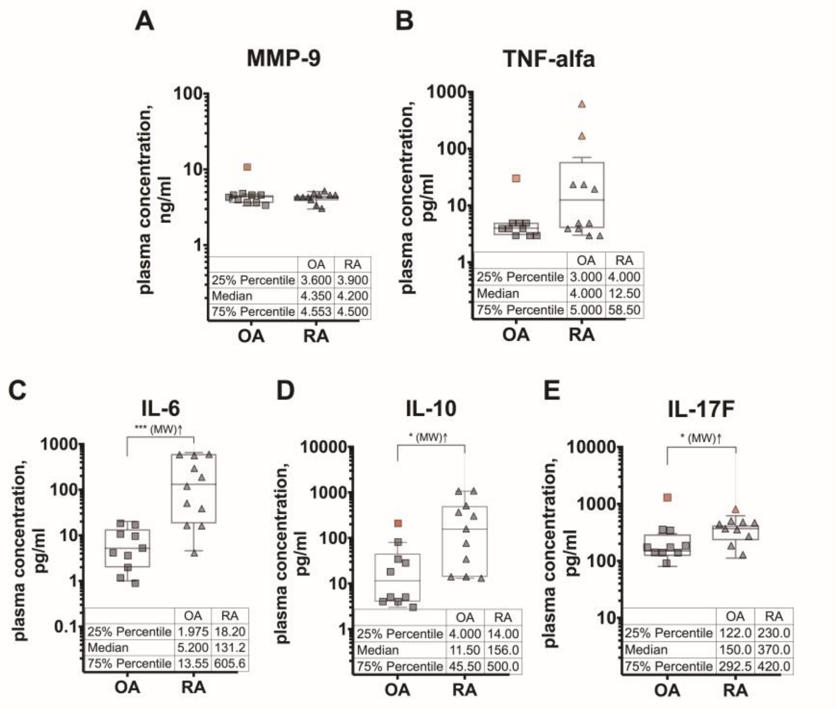

Figure S2. Levels of MMP-9, TNF-α, IL-6, IL-10, and IL-17F in the peripheral blood plasma from rheumatoid arthritis (RA) (n=15) and osteoarthritis (OA) (n=10) patients. Distribution of the values among the individual patients is shown. Data are presented as the overlapping scatter and box-and-whiskers plots with the medians and IQR (25% percentile and 75% percentile); red symbols show the outliers; * $p < 0.05$, *** $p < 0.001$, MW—Mann–Whitney test.
